# Aristotle, Buddhist scripture and embryology in ancient Mexico: building inclusion by re-thinking what counts as the history of developmental biology

**DOI:** 10.1242/dev.192062

**Published:** 2021-02-02

**Authors:** John B. Wallingford

**Affiliations:** Department of Molecular Biosciences, University of Texas at Austin, 2401 Speedway, Patterson Labs, Austin, TX 78712, USA

**Keywords:** Asia, History, Inclusion, Latin America

## Abstract

It has not gone unnoticed in recent times that historical writing about science is heavily Eurocentric. A striking example can be found in the history of developmental biology: textbooks and popular science writing frequently trace an intellectual thread from the Greek philosopher Aristotle through 19th century embryology to 20th century genetics. Few in our field are aware of the depth and breadth of early embryological thinking outside of Europe. Here, I provide a series of vignettes highlighting the rich history of embryological thinking in Asia and Latin America. My goal is to provide an entertaining, even provocative, synopsis of this important but under-studied topic. It is my hope that this work will spur others to carry out more thorough investigations, with the ultimate goal of building a more inclusive discipline.

## Abstract (precis)

**Summary:** This Review highlights the rich history of embryological thinking in Asia and Latin America, challenging the European-centric view of the history of scientific investigation and discovery in developmental biology.

## Introduction

Last year marked the 80th anniversary of the founding of the US-based Society for Developmental Biology, an anniversary that was exciting for marking tremendous advances, both technical and intellectual. Although 80 years is a long time, the central question of how embryos develop has been consistently pondered for well over 2000 years.

In a recent essay, I argued that everyone is a developmental biologist (Wallingford, 2019b), contending that developmental biology is fundamental to the human experience. My contention is provocative, and of course my use of ‘developmental biologist’ here is far broader than is traditionally employed (Crowe et al., 2015; Hopwood, 2019). Nonetheless, I believe this conceit is useful, even important, because it will help modern developmental biologists to understand that our scientific curiosity is related to the far more ancient and universal human pondering of how each of us comes into being. Moreover, although embryos have been contemplated and even directly observed for millennia, the same cannot be said for mitosis or the immune response. For these reasons, I believe that developmental biologists have a particularly pressing obligation to understand the history of not just our modern discipline but also of the pondering of embryos generally.

Indeed, Aristotle is sometimes considered the ‘first’ developmental biologist, and his book *On the Generation of Animals*, written in the late fourth century before the Common Era (BCE), dominated European thinking about embryos for over 1500 years. More recently, Charles Darwin took time to explicitly discuss embryological considerations in *On the Origin of Species* (Darwin, 1859). In the intervening years, William Harvey, famous for his discovery of blood circulation, also studied embryos, striking a major blow against the notion of spontaneous generation in the process. As did Robert Boyle, of Boyle's Law in chemistry; his report on holoprosencephaly in a newborn horse is a fascinating read (Boyle, 1665). Writing of the cyclopic head, which he had ‘hastily and rudely cut off’, he then ‘caused it to be put into a vessel and covered with spirit of wine’. Sounds like a developmental biologist to me!

Many reading this essay will have heard some or all of these tales, in part because they frequently appear in modern textbooks and popular writing about developmental biology. But it is also the case that nearly a century of comprehensive histories have presented these examples to illustrate the evolution of modern developmental biology and embryology from earlier studies of ‘reproduction’ and ‘generation’ (e.g. Gilbert, 1994; Hopwood et al., 2018; Laubichler and Maienschein, 2007; Maienschein, 2014; Meyer, 1939; Needham and Hughes, 1959). Many developmental biologists will also have heard about more recent forebears, such as Thomas Hunt Morgan, Hans Spemann and Hilde Mangold, or Karl Ernst von Baer. What they are almost certain not to have heard, however, is a serious consideration of early embryological thinking from the non-European world.

**Fig. 1.**
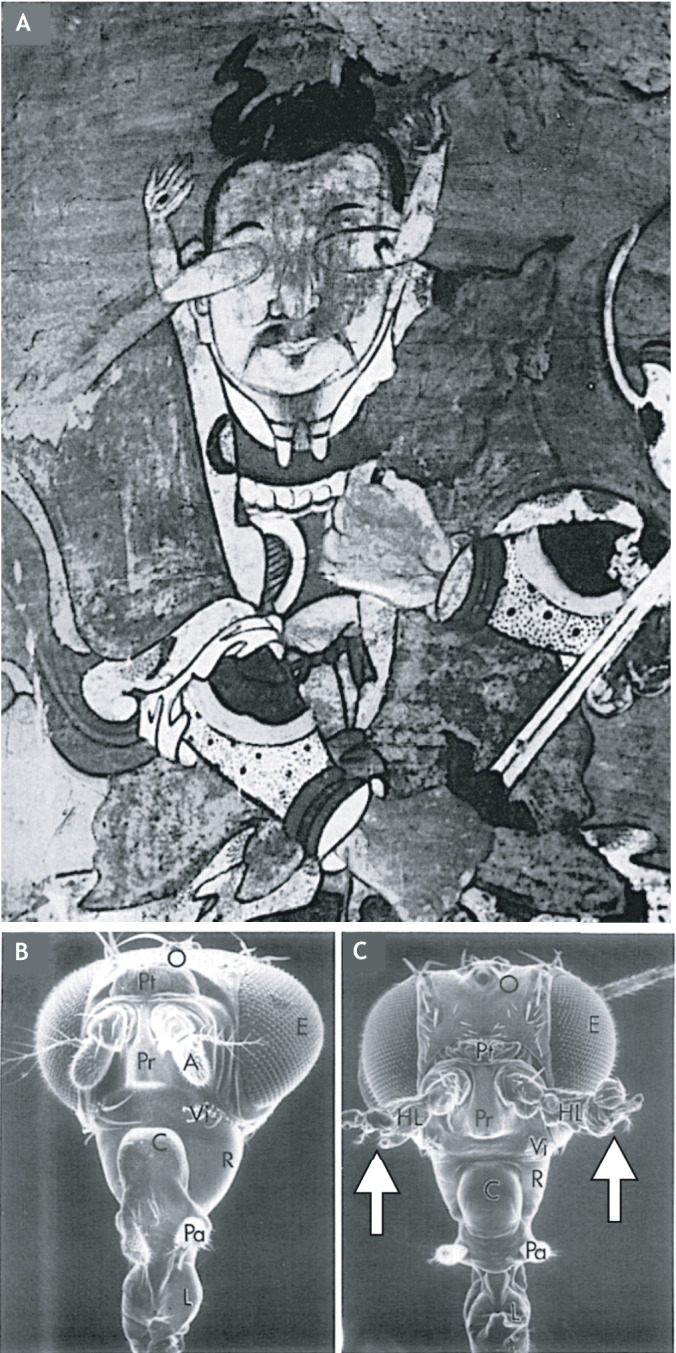
**Homeotic mutants in Ancient China? (**A) The historian and embryologist Joseph Needham photographed this fresco depicting a guardian deity on a wall in the roughly 2000-year-old Dunhuang Cave Temples in Gansu, China. Needham speculated that the arms growing out of the eyes may reflect the painters’ observation of heteromorphosis in crustaceans, which frequently regenerate an incorrect appendage after amputation. Personally, I prefer to think that the painter observed a homeotic mutant in an arthropod. (B) A scanning electron micrograph of a normal *Drosophila melanogaster* (Postlethwait and Schneiderman, 1971). (C) In the homeotic mutant, *Antennapedia*, the antennae are replaced by ectopic legs (arrows). The image in panel A is reproduced with permission from Needham and Hughes, 1959; the images in panels B and C are reproduced with permission from Postlethwait and Schneiderman, 1971.

The definitive 20th century history of embryology was written by the Cambridge polymath Joseph Needham, a pioneering embryologist in his own right (and also an ardent communist with a famously open marriage) (Winchester, 2008). However, Needham's encyclopedic *A History of Embryology* dedicates only a few pages to Asian embryology (Fig. 1) (Needham and Hughes, 1959), which is surprising since he went on to great fame for his efforts in documenting the history of Chinese science (Winchester, 2008). Moreover, none of the subsequent comprehensive histories of the field have addressed the issue either. I believe this oversight must be corrected: there is, in fact, a very rich history of early non-European embryological thinking, though to date it has been addressed primarily by religious scholars and art historians.

I have argued for the power of storytelling as a corrective measure for what many feel is a current underappreciation of developmental biology (Gilbert, 2017; St Johnston, 2015; Wallingford, 2019b; Zon, 2019). What follows, therefore, is not so much an effort in documenting the history of developmental biology per se, but rather is a series of vignettes highlighting the breadth of historical thought about embryos. My primary objective is to inform, entertain, and perhaps inspire working developmental biologists, and in the process provide additional context for connecting their work to the world. With any luck, though, I will also spur historians of science to explore these issues further.

## Embryos in early Asia: the transmigration of consciousness and an accurate description of human development

It will probably surprise most developmental biologists to learn that there is an immense record of ancient thought about embryos in Asia. Although ancient Greek scholars, culminating with Aristotle, clearly possessed a deep interest in embryonic development, the written record of embryological thought in ancient Asia is in some ways even more impressive. As was the case in ancient Europe, early Asian embryological thinking long predated epistemological distinctions between science, philosophy and religion. Indeed, the transmigration of the consciousness from the dead to the living is a central tenet of Buddhism and it is clear that some early Buddhist scholars pondered embryonic development from this standpoint. In fact, discussions of embryogenesis – and speculation about its mechanisms – can be found in Asian religious scriptures dating back several centuries BCE. Importantly, however, there is also a clear record of secular embryological thought in Asian medical or scientific settings that is very nearly as old. For an outstanding general overview, I refer the reader to Andreeva and Steavu (2015b).

One of the oldest extant Asian texts describing embryonic development is the *Garbhopaniṣad*, which is one of the Upanishads – a series of Indian religious texts that collectively date to the middle of the first millennium BCE. This text provides a brief description of the process of human development over time, stating that the embryo is ‘like water in the first night, in seven nights it is like a bubble, at the end of half a month it becomes a ball. At the end of a month it is hardened, in two months the head is formed…’ (translated in Aiyar, 1914). The text further makes it clear that, even at this early date, the author(s) were able to link embryogenesis to congenital anomalies, stating that limb deformities result from eclipses of the sun and moon, and dwarfism and achondroplasia from parents with anxious minds (Aiyar, 1914). This latter notion – that the thoughts of parents, especially mothers, could influence embryonic development – was more thoroughly developed in later Asian writings and was also prevalent throughout Europe and the Americas until well into the modern age (Garland-Thomson, 1997).

By the early centuries of the Common Era (CE), consideration of the embryo in India began to differentiate into ‘medical’ and ‘religious’ writings. Although both categories attempted to provide explanations for conception and embryogenesis, medical documents such as the *Suśrutasaṃhitā* and *Carakasaṃhitā* did so dispassionately, focusing on promoting healthy pregnancies (Kritzer, 2009; Selby, 2005). By contrast, the contemporaneous *Garbhāvakrāntisūtra* had a clearly religious intent, advancing Buddhism's First Noble Truth, that all life is suffering, even *in utero* (Kritzer, 2009). Translated and extensively analyzed by Robert Kritzer, this scripture repeatedly describes the embryo as ‘very hot, very unbearable, oppressed, and pained, having a consciousness with the sole flavor of suffering’. The text slides into what has been called the ‘ascetic misogyny’ of some early Buddhist writings, repeatedly describing the womb as a ‘filthy, putrid blazing bog’ (Kritzer, 2014).

What is striking, however, is that the overtly religious *Garbhāvakrāntisūtra* is by far the more accurate document. Indeed, it may well be the single most accurate description of human development from the ancient world. However, although it was described briefly by the developmental biologist David Kimelman (Kimelman, 2018), this fact appears not to have come to the attention of science historians. As it happens, the *Garbhāvakrāntisūtra* carefully describes each week of human gestation for 38 weeks (Kritzer, 2014). The text describes the embryo in the first three weeks as being like ‘the liquid part of yogurt… hardened butter… a roundworm’. In the fifth week appear ‘the upper arm signs of the two upper arms, the thigh signs of the two thighs, and, fifth, the head sign of the head’. Here, the words translated by Kritzer from Chinese and Tibetan as ‘signs’ (which embryologists may have called ‘anlagen’) are commonly used terms that also mean ‘omen’, providing a glimpse of scholars setting the problem into words.

**Fig. 2.**
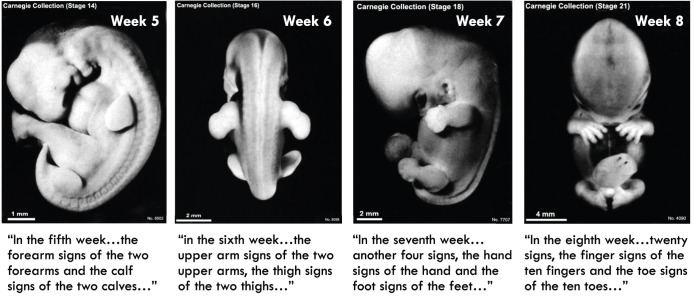
**The *Garbhāvakrāntisūtra* prefigured the Carnegie stages by 2000 years.** Panels depicting Carnegie stages of human development (O'Rahilly and Müller, 1987) with accompanying passages from the *Garbhāvakrāntisūtra* translated by Robert Kritzer (Kritzer, 2014) provided below each panel. David Kimelman has previously made a similar comparison (Kimelman, 2018). Images obtained from https://embryology.med.unsw.edu.au/embryology/index.php/Main_Page.

Perhaps the most striking descriptions in the *Garbhāvakrāntisūtra* are of the fifth through eighth weeks of human development, where the text describes the development of first the limbs, then the hands and feet, and then of the fingers and toes. Even the most careful comparison to the Carnegie stages of human embryogenesis reveals a startling accuracy to this ancient treatise (Fig. 2). By contrast, while the contemporaneous Indian medical texts lack any discussion of suffering or commentary on the womb, their descriptions of human development are far less precise, with the progression of development being described far slower than in reality. For example, the *Suśrutasaṃhitā* places limb development in the third month (Kritzer, 2009).

That said, the medical texts and the *Garbhāvakrāntisūtra* do agree on several important points. First, all state that the embryo arises from the mingling of semen and female reproductive fluids (likely menstrual blood), together with an additional ‘cause’ (Kritzer, 2014), repeating ideas set down much earlier in the *Garbhopaniṣad* (Aiyar, 1914). This assertion is interesting for its striking similarity to notions espoused by ancient Greek writers (Needham and Hughes, 1959). Another aspect shared by all of the Indian sources is that complexity and form in the embryo are built up gradually, stage by stage (Kritzer, 2014). A similar developmental scheme was termed ‘epigenesis’ by early European embryologists, for whom it stood in contrast to the idea of preformation (i.e. the homunculus), although Kritzer notes that he found no evidence for preformationist ideas in either religious or medical embryologies in early Asia (Kritzer, 2014). Consistent with the idea of ‘epigenesis’, the early Indian thinkers developed a largely consistent set of Sanskrit terms to describe this progressive development. Like ourselves, they used both a catchall term for the embryo, garbha, as well as more specific terms to describe particular stages. So, rather than the morula, blastula, gastrula and neurula, the authors used the Sankrit terms kalala, arbuda, peśī and praśākhā (Andreeva and Steavu, 2015a; Suneson, 1991).

Such thinking was in no way limited to India and, in fact, the earliest clearly medical Asian text on embryology was found in China. This text, the *Taichanshu*, is among the many medical writings found in the Mawangdui Caves, which are considered to have been sealed no later than 168 BCE (Andreeva and Steavu, 2015a). This scripture was intended to promote healthy pregnancy outcomes, and its month-by-month description of development is accompanied by precise recommendations for expectant mothers (Harper, 1998; Wilms, 2018). By the third century CE, the *Garbhāvakrāntisūtra* had been translated into Chinese (Kritzer, 2014). However, perhaps owing to the overtly religious message of the *Garbhāvakrāntisūtra*, later Chinese treatises such as the *Qianjinfang* describe embryology in a similar manner to that used in the less-accurate earlier Indian medical texts (Andreeva and Steavu, 2015a; Cook and Luo, 2017; Wilms, 2005).

Crucially, however, the Chinese scholars did provide a huge innovation in embryological thinking: they drew pictures. Like most of the Chinese discussions of embryology, the *Taichanshu* had an obstetric point of view, so it was accompanied by sketched outlines of a female form and information for predicting the child's future (Harper, 1998). Even more sophisticated is the *Ishimpo*, a 10th century Japanese compendium of Chinese medical writings. Later versions of this treatise contain not only images of a mother's body across each month of pregnancy (with guidance for the application of acupuncture), but also depictions of the developing embryo (Wilms, 2005, 2018). This series does not simply depict growth, but rather captures a developmental progression, showing a small, round embryo during the first and second months, and a more human-like fetus in the third and fourth, which then grows substantially through the rest of pregnancy (Wilms, 2005).

Ultimately, the original dates for the illustrations in the *Ishimpo* are unknown, but they are not later than the 12th century, and could be earlier (Wilms, 2005, 2018). The historian Janina Wellmann has argued that ‘the history of developmental thinking cannot be written without attending to the forms and conventions employed to visualize development’ (Wellmann, 2017). Indeed, the chronology of chick embryos illustrated by Fabricius in 1621 and Soemmerring's images of successive stages of human embryos in 1799 have been the subjects of intense discussion by historians (e.g. Ekholm, 2018; Hagner, 1999; Hopwood, 2000; Wellmann, 2017). However, the all but unknown *Ishimpo* may very well be the oldest known chronological visualization of embryogenesis in history. Thus, although the later European illustrations were more accurate and systematic, there is no question that ancient Asian embryological thought developed a high degree of sophistication. It deserves further study.

## Ontogeny and phylogeny in early Tibet

The timing of the *Ishimpo* provides an excellent point of reference for our next story of embryological thought in Asia. Around the same time, perhaps 800 years ago, Tibetan scholars initiated a wide-ranging debate on the precise sequence of human embryonic development (Garrett, 2005; Gyatso, 2015). As early as the 9th century, the *Garbhāvakrāntisūtra* had been translated into Tibetan from the Sanskrit and from earlier Chinese translations (Kritzer, 2014). And yet, several Tibetan scholars of the day described versions of embryonic development that were far less accurate, seeming to adhere more closely to the *Carakasaṃhitā* and *Suśrutasaṃhitā* descriptions discussed above (Garrett, 2005, 2009). Still others described entirely novel sequences. Most fascinating is the work of the Tibetan scholar Longchempa in the 14th century. Among other things, his text correctly places the development of the limbs around the eighth week of gestation (Garrett, 2005), but what interests me the most is how he defines the stage before the limbs are developed: he refers to it as the ‘fish’ stage. The seventh week is then termed the ‘tortoise’ stage and, as limbs develop in the eighth week, Longchempa refers to the ‘frog’ stage. Similar, though less detailed, progressions were described by other authors as early as the 12th century (Garrett, 2005; Norbu, 1987).

**Fig. 3.**
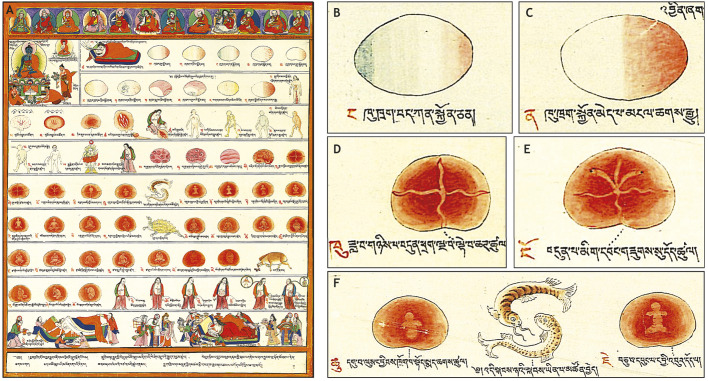
**The stages of human development according to the *Four Tantras*.** (A) The entire Panel 5 of Sangye Gyamtso's Illustrated *Four Tantras* (Gyamtso et al., 1992). (B) Though reminiscent of modern images of maternal gradients, this image is said to depict ‘semen and blood damaged by phlegm’. Like the Greeks, Tibetans believed the embryo to arise from mingling between semen and menstrual blood. The *Four Tantras* describes several conditions whereby conception will fail; this is one of them. (C) Image depicting ‘semen and menstrual blood with no defect, likely to lead to conception’ (i.e. the normal kalalam). (D,E) Later stages – depicting the development of ‘channels’ – resemble cleavage-stage amphibian embryos. (F) Still later stages of development are symbolized as the ‘fish’ stage. Images reproduced with permission from Gyamtso et al., 1992, Serindia Publications.

Now, it is important to note that the fish and the tortoise also happen to be successive incarnations of the Indian god Vishnu, and it has been argued that the haphazard, even whimsical, variability of Tibetan embryological treatises can be explained by ‘literary aesthetics’ (Garrett, 2005). However, in light of the stunning accuracy of far earlier Asian descriptions of the human embryo, it seems possible that the variability could instead (or at least in part) reflect deeply religious scholars trying to interpolate centuries of thought and tradition with their own observations of very small objects. Maybe these authors had looked at early human embryos and thought they looked like fish. After all, that's more or less what Ernst Haeckel did in 19th century Europe. We now know that Haeckel's flawed but persistent argument that ‘ontogeny recapitulates phylogeny’ was developed with the help of infamously inaccurate drawings and specious interpretations (Hopwood, 2015; Needham and Hughes, 1959). But, it is still a fact that early mammalian embryos, including human embryos, bear such a similarity to the adult forms of more evolutionarily basal vertebrates that the notion was held by very serious scholars for over a century (Gould, 1977). It is interesting, then, that when the Tibetan illustrators Lhobrag Norbu Gyamtso and Lhasawa Genyen depicted the chronological progression of human development in 1670, they chose to place the ‘fish’ stage precisely before the formation of limbs (Fig. 3A), at a time when the pharyngeal arches of the still-limbless human embryo do resemble the gills of fish.

These talented medical illustrators were recruited by Sangye Gyamtso, who at the behest of the Fifth Dalai Lama was revising the foundational document of Tibetan Medicine, *The Four Tantras.* Sangye Gyamtso felt that ‘presentation [of concepts and concrete objects] in special paintings would be of benefit to a deep understanding’ (Gyamtso et al., 1992). His *Four Tantras* was therefore accompanied by 77 paintings, the fifth of which conveyed the author's and artists’ understanding of the chronology of human development (Fig. 3A-F). Any developmental biologist will see some very familiar images here. Stunningly, many of the images could have been lifted from modern diagrams of the maternal gradients in *Drosophila* or the Par proteins in *Caenorhabditis elegans*! Of course, the artists could not have seen such things, and although it will be tempting to wonder if these ancient scholars pondered something akin to ‘polarized maternal determinants’, the accompanying text does not support that view. Nonetheless, it clearly does reflect scholars thinking very hard about embryos (Dakpa, 1993; Gyamtso et al., 1992).

It is striking, then, that this Tibetan chart appears rarely to have been discussed in the context of science history, even while the challenge of visually representing the dynamic, three-dimensional problem of embryology has been considered so extensively (e.g. Ekholm, 2018; Hopwood, 2000, 2005; Wallingford, 2019a; Wellmann, 2017, 2018). Even more striking is the early date of this Tibetan work, which is largely contemporaneous with that of Fabricius. In fact, Sangye Gyamtso's decision to engage skilled illustrators prefigured the widely heralded similar decision of Ignaz Döllinger by 150 years: Döllinger cajoled his student Christian Pander to recruit the engraver Eduard D'Alton, and together with von Baer, they provided the first truly systematic illustrated descriptions of embryonic development, including early efforts to convey information in three dimensions. In so doing, they initiated not only modern embryology but also the visualization schemes we still use – and struggle with – today. So, might we claim the early Tibetan scholars as developmental biology forebears? Clearly, more research would be required to do so. But if we consider that possibility, then perhaps the debates among early Tibetans could provide a useful parallel when considering the debates among Renaissance and Enlightenment Europeans as they struggled to understand (and visualize) the embryo. Perhaps such a broader view of the struggle would help us to understand how culture influences the thinking of scholars who study embryos. Such an understanding might also be useful as we grapple with modern developmental biology and its implications.

## From garbha to goldfish: ‘model organisms’ in early Asia

Asian texts clearly indicate a rich history of thinking about the human embryo, but what of ‘animal models’? Clearly, the concept of ‘model organisms’ is a very modern one, but I feel this tongue-in-cheek reference is apt, as many authors are quick to invoke Aristotle for the origin of comparative embryology, and many point out that European thinkers such as Étienne Geoffroy Saint-Hilaire used animal embryology in their attempts to understand human congenital anomalies. I argue that we should likewise consider the extent to which early Asian scholars pondered animal embryos to further their understanding of human development.

The Pali Canon is an important collection of Buddhist scriptures dating to sometime near the turn of the Common Era, and its passages make it clear that the authors were keen observers of animal reproduction. These early texts also make a clear distinction between viviparous mammals and oviparous reptiles and birds. Moreover, they define yet another class, ‘produced from rotting fish, rotting corpses, rotting rice, or a dirty pond’ (Boisvert, 2000), a passage suggesting an early struggle with the idea of spontaneous generation. Such thought can also be found in Asian writings from millennia later (Chen, 1956; Needham, 1956), reflecting the similarly long struggle with the concept in Europe (Needham and Hughes, 1959). Interestingly, the Pali Canon authors use the same term, kalalam, to refer to early embryos in all classes, indicating a conceptual linkage between animal and human embryos (Boisvert, 2000).

In 17th century Tibet, this linkage may have become more concrete. Indeed, in Gyamtso's *Four Tantras* the image described as depicting the normal human kalalam is strikingly reminiscent of an asymmetrically pigmented amphibian egg (Fig. 3C). Now, that might seem like the wishful thinking of the aging amphibian embryologist writing this essay, but a later image in the *Four Tantras* series even more obviously resembles a four-cell frog embryo, with subsequent images reminiscent of cleavage-stage frog embryos (Fig. 3D,E). The text accompanying these particular panels states that they depict the formation of ‘channels’ (Dakpa, 1993; Gyamtso et al., 1992). Now, in a contemporaneous commentary on the *Four Tantras*, the authoritative Lodrö Gyelpo Zurkharwa explicitly discussed development of the channels, repeating a description of their three-fold symmetry from older Buddhist treatises (Gyatso, 2015). Why, then, did his artist colleagues choose these particular images, with the obvious four-fold symmetry (Fig. 3D)?

One possibility is that, like so many biologists past and present, these scholars looked at an animal embryo and attempted to extrapolate what they saw to humans. Indeed, some Tibetan frog species produce embryos over 2 mm in diameter, making them readily observable, even without magnification (e.g. Chen et al., 2013; Lu et al., 2016). But, as entertaining as this line of thinking may be, scholarship demands us to acknowledge that just because those images look like frog embryos to a modern developmental biologist, it is not actually evidence of the artists’ intent. Again, more research is warranted.

So, what about direct written descriptions of animal embryos in early Asia? In the West, artificial incubation of chicken eggs for agriculture provided Aristotle with ample material for his early studies (Needham and Hughes, 1959). Though similar practices were common in China by the second century BCE (Landauer, 1961), the more relevant factor for Asian embryology might have been the widespread pursuit of aquaculture (Chen, 1956; Smartt, 2008). Like so much of Asian embryology, the Chinese tradition of aquaculture has its origins in Buddhism. In his exhaustive review of goldfish aquaculture in China, Chen writes: ‘As one of the chief tenets of Buddhism is abstention from taking life, we began, ever since the religion was introduced into China, to set free live animals.’ This led to the tradition of maintaining ‘ponds of mercy’ into which fish could be released by the faithful.

By the Ming Dynasty in the 1500s, the rearing of goldfish transitioned from ponds to aquariums, facilitating closer observation of their biology (Chen, 1956; Smartt, 2008). This tradition of aquaculture facilitated artificial selection, which in turn led to the generation of a wide array of goldfish, modifying not only coloration, but also morphology, with breeds displaying bizarre alterations of the tail, eyes and head (Omori and Kon, 2018; Ota and Abe, 2016). Such selection may have begun over 1000 years ago (Smartt, 2008), but Chen's translations make clear that by the 16th century, there was a pointed interest (and an extensive literature) in the development of these fish. For example, Lung Tu wrote in 1592 that: ‘I have often wondered at the multifarious transformations of the goldfish in color and form. I have consulted a wide range of books dealing with fishes’. In 1630, Hsiang-Chin Wang wrote: ‘Examine the water-grass in the light of the sun; if you find little crystalline granules about the size of millet, they are the eggs.” Finally, in the early 1600s, Shen noted: “The kind with gold and silver eyes, or double rings, or nine tails, are merely good-looking novelties… like men with the abnormal growth of a finger.” This line may suggest an author considering the parallels between animal and human development. (Note: all translations are from Chen, 1956).

In light of this very long history, it is perhaps not surprising that the goldfish came to be exploited by modern Chinese embryologists. In her excellent recent study of Chinese embryology in the mid-20th century, the historian Lijing Jiang highlights the work of Zhu Xi and Dizhou Tong, and how their work was shaped by their socialist surroundings (Jiang, 2018). Xi contributed the first comprehensive modern studies of the early development of the goldfish, but Tong is the more interesting character, in my view. Now, it is possible that I am biased by Tong's ahead-of-its-time work on the polarization of ciliary beating in amphibians (Tung and Chang, 1949; Tung and Tung, 1940), a topic near and dear to my heart (e.g. Park et al., 2008). And it is also possible that I am impressed by his clear thinking on cell movements during morphogenesis (Jiang, 2018). Or it may simply be that if we are looking for good stories to tell, it's hard to top the one about a man who: 1) after being blacklisted had unknowingly avoided arrest by leaving China for a research stint at the Marine Biological Laboratory in Woods Hole, MA, USA and 2) was the first person to clone a fish. Naturally, it was a goldfish (Jiang, 2018).

## Art meets embryology in ancient Mexico

Moving away from Asia, let us now consider the possibility that the oldest realistic images of embryos anywhere were actually made in southern Mexico. That is the contention of Carolyn Tate and her colleagues who have studied 3000-year-old statues made by the pre-literate Olmec people. Their analysis reveals that dozens of statues display head-to-body proportions of between 1:3 and 1:4, appropriate for fetuses between 12 and 30 weeks (Tate and Bendersky, 1999). Even more striking are the statues that have embryonic proportions but also display paddle-like limbs and distorted craniofacial features, resembling early human embryos as they undergo limb and craniofacial morphogenesis (Tate, 2012). In addition, many of these statues clearly seem to depict craniofacial anomalies and neural tube defects, which must have been as prevalent then as now (Bendersky, 2000; Tate and Bendersky, 1999).

Now, these are interpretations of objects that were created by a civilization that left no writing and disappeared millennia ago. It therefore bears noting that, although some Mesoamerican scholars accept the view that these are statues of human embryos, it is not widely embraced. In one case, however, there is no ambiguity: a sculpture unearthed in the Mexican city of Oaxaca and dated to roughly 1400 BCE clearly depicts a human fetus, as the tiny figurine was found inside a cavity in the lower abdomen of a larger, clearly female, figurine (Marcus, 1998). Some 3000 years later, when the Franciscan Friar Bernardino de Sahagun visited New Spain just after the conquest of the Aztecs by Cortés, he found a highly sophisticated culture of obstetrics and midwifery (Schwartz, 2018). The Aztecs had a literal pantheon of gods and goddesses dedicated specifically to fertility, gestation and childbirth, so it seems reasonable to consider more carefully the embryological thinking of ancient Mesoamericans.

Let us now consider the axolotl. This star of current regeneration research holds a unique place among modern model animals. Indeed, although we know little, if anything, about the ancients’ thinking on *Drosophila* or *Danio*, the axolotl has been written about for centuries (Reiß et al., 2015), and even the Aztec god Xolotl is well-known to have once transformed into an axolotl (Smith, 1989; Wanderer, 2018). Moreover, axolotls were an important food source for early Mesoamericans. Sahagun called them the ‘food of gentlemen’, but there is abundant evidence that early Mesoamericans ate not only axolotls, but also frogs (Kennedy, 1982; Tate, 2010; Wanderer, 2018). Coupled to the fact that axolotl embryos are very large and their development easily visible to the naked eye, this consistent proximity between people and amphibians makes it possible to consider that early Mesoamericans did actually observe developing embryos. Strikingly, several authors have noted that certain Olmec carvings resemble tadpoles (Kennedy, 1982; Pohorilenko, 1996). The fact that many such carvings are also engraved with images resembling human embryonic faces may suggest an artist's conceptual linkage of tadpoles and human embryos (Tate, 2012).

I want to stress again that the meaning of Olmec carvings and the intent of their creators remain much debated. Just the same, in light of the well-described record of ancient embryological thought in Europe, and the vast but vastly underappreciated record of similar thinking in ancient Asia, similarly complex contemplation very likely occurred in ancient America (and Africa, and Australia). The topic clearly deserves more attention, especially in the context of my contention that developmental biology is embedded in culture itself. For example, the early intersection of embryos and religion so abundantly clear in Asia and Europe may have been just as prevalent in ancient Mexico. Indeed, a violent, sometimes deadly, ritual ballgame was a ubiquitous feature of many early Mesoamerican cultures, and was central to their world view. It may be fitting, then, that many of the Olmec fetus statues also happen to be wearing helmets (Tate, 2012). Ultimately, in the intricate web woven by science, religion and culture, the embryo touches all strands.

## A history of embryology in Mexico

Because embryological thought is both ancient and universal, we must then consider how regional culture may impact the trajectory of regional embryological thinking. For a preliminary contemplation of this issue, let us return to Oaxaca, in southern Mexico. Fascinatingly, this city produced not just the early depiction of embryological thinking, as discussed above, but also what may be the earliest written description of embryological thinking in the Western Hemisphere.

In an outstanding study of reproductive issues in colonial Mexico, Nora Jaffary describes her discovery in the *Biblioteca Francisco de Burgoa* in Oaxaca of documents clearly indicating local contemplation of both human birth defects and ‘animal models’ (Jaffary, 2011). What she found was a single handwritten page describing the birth of conjoined twin girls in Oaxaca in 1741. This description was decorated by a sketch of the girls. More interestingly, this single page was tucked into a copy of a 1651 treatise by the naturalist Francisco Hernandez precisely at the page where he describes the birth of a two-headed calf. The handwritten note, by an unknown author, acknowledges these similarities, then goes on to also describe a four-winged, four-legged chicken observed at the monastery housing the library (Jaffary, 2011).

This was not an isolated case, as over 50 cases of congenital anomalies were carefully described in the *Gazeta de Mexico* in the last quarter of the 18th century alone (Jaffary, 2011). In fact, we can find widespread discussions of embryological thought going back at least to the 17th century in both Mexico and Guatemala (Few, 2009; Jaffary, 2011). Such descriptions became increasingly sophisticated, appearing in the *Periodico de Academia de Medicina de Mexico,* which is the precursor of the present *Gaceta Medica de Mexico*, the oldest continuously published scientific journal in the Americas (Chico-Ponce de León and Castro-Sierra, 2007). Despite the volume and sophistication of this work, it seems yet to have been examined by science historians.

Consider, for example, the human birth defect holoprosencephaly, which produces cyclopia and holds a prominent place in the ancient history of human pondering of the embryo (Cohen, 2010). The first ‘scientific’ description of holoprosencephaly may be that of Robert Boyle, although the first scientific discussion of human holoprosencephaly is attributed to Eller in 1755, and Isidore Geoffroy de Saint-Hilaire's work from the 19th century has been much discussed (Cohen, 2010). However, what is not widely known is that Juan Nepomuceno de Miranda described a case of holoprosencephaly in a Mexican medical text in 1785 (Chico-Ponce de León and Castro-Sierra, 2007). This description is accompanied by a detailed woodcut, clearly depicting a median proboscis above the single eye, a hallmark of severe cyclopia caused by loss of Hedgehog signaling (Chiang et al., 1996).

**Fig. 4.**
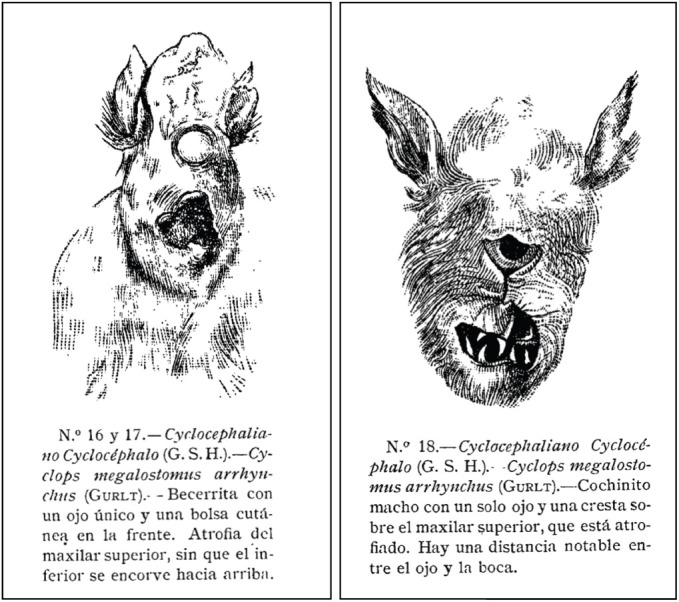
**19th Century Mexican depictions of holoprosencephaly.** Juan Nepomuceno de Miranda described a case of holoprosencephaly in a Mexican medical text in 1785 (Chico Ponce De Leon and Castro-Sierra, 2007). Roughly a century later, in his catalog of the collection in the National Museum of Mexico, Ramirez (1896) presented the specimens illustrated here, a calf (left) and a piglet (right). In the catalog, these were juxtaposed with images of human holoprosencephaly. Images reproduced with permission from the Benson Latin American Collection, LLILAS Benson Latin American Studies and Collections, The University of Texas at Austin, USA.

By the mid-19th century, Mexican teratology had achieved a high level of sophistication (Jaffary, 2020). This was a time when leading institutions across the world assembled large anatomical collections with a focus on congenital anomalies (Hagner, 1999); the Mütter Museum in Philadelphia is a prominent American example (McLeary, 2000). Mexico was no exception. In 1895, the Mexican Museo Nacional assembled its own ‘Salon de Anomolias’ and tasked the physician Roman Ramirez with drafting the accompanying catalog. In his summary of key principles, he notes that ‘teratology cannot be thoroughly studied without previously knowing embryology’ (Ramirez, 1896). Fascinatingly, this collection was dominated not by human specimens, but by animals (Fig. 4), all of which are carefully classified using the system developed by Geoffroy Saint-Hilaire.

It is notable, then, that at roughly the same time, the work of European teratologists was being used to set the stage for the rise of what we now unfortunately call ‘freak shows’ (Garland-Thomson, 1997). The boorish Mexican-themed display of ‘The Aztec Lilliputians’ in London (Aguirre, 2003; Richards, 1994) provides a striking counterpoint to actual Mexican teratology of the day. Indeed, Jaffary has argued that, by directly comparing congenital anomalies in humans and animals, 19th century Mexican teratology appeared intent to emphasize ‘its normalcy within – rather than its aberration from – the spectrum of organic creation’ (Jaffary, 2020).

Ultimately, the way we understand embryos – both as scientists and merely as human beings – is impacted by the cultures in which we live. Thus, the thinking of early Mexican teratologists was informed at some level by ancient Mesoamerican thinking (Few, 2009), just as European scholars were informed at some level by Homer's cyclops. Given the tremendous, yet largely ignored, medical toll of congenital anomalies (Wallingford, 2019b) and our often fraught understanding of their place in society (Garland-Thompson, 2017), perhaps a broader examination of their history in different cultures will be useful.

## Conclusions and perspectives

It is ironic that the 20th century's foremost scholar of the history of embryology, Joseph Needham, was also its foremost scholar of Asian science, yet he wrote almost nothing of Asian embryology. It is interesting to note, though, that he was not a trained historian. He was an embryologist, and a notable one. His enormous *Chemical Embryology* (in several volumes, covering roughly a linear foot of shelf space) was a decades-ahead-of-its-time attempt to understand the molecular basis of development (Needham, 1931). And, though he ignored Asian embryology, the central thesis of his decades-long study of Chinese science was actually that ancient science in Asia far surpassed that in the West (see Winchester, 2008). It is a particular shame, then, that the very idea that early non-European thinking can be considered ‘science’ has been frequently debated. Indeed, another great developmental biologist, Lewis Wolpert, devoted a whole chapter to arguing for a single Western origin for science in his otherwise excellent 1992 contemplation, *The Unnatural Nature of Science* (Wolpert, 1992).

Like Needham and Wolpert, I am not a historian; I am a working developmental biologist considering the state of our field and its place in the world. Accordingly, I should make it clear that I do not argue (and would be not qualified to argue) that any of what I have discussed here relates to the intellectual evolution of the modern discipline of developmental biology. But I do believe it opens the door to a better understanding of how humans think about embryos, and that is the very essence of our craft.

I also know this: for 30 years, I have been inspired by stories of ‘developmental biologists’ across the ages. As we work to expand the field and keep it relevant, we must constantly work to inspire our students, all students. Thus, if we mention Aristotle in developmental biology textbooks, and we do (Gilbert and Barresi, 2020; Wolpert et al., 2015), we should also mention the *Garbhāvakrāntisūtra.* If we weave Aristotle into popular science writing about developmental biology, and again, we do (Leroi, 2005, 2014; Wolpert, 2011; Zernicka-Goetz and Highfield, 2020), it is time to find a place for the *Ishimpo*. If we consider Fabricius’ artwork, we should consider Gyamtso's. When we teach Geoffroy Saint-Hilaire, let us also teach Ramirez. There is a whole world of developmental biology history waiting to be told. So, let's go to work.
